# Multiple sources of water preserved in impact glasses from Chang’e-5 lunar soil

**DOI:** 10.1126/sciadv.adl2413

**Published:** 2024-05-10

**Authors:** Chuanjiao Zhou, Bing Mo, Hong Tang, Yaya Gu, Xiongyao Li, Dan Zhu, Wen Yu, Jianzhong Liu

**Affiliations:** ^1^ Center for Lunar and Planetary Sciences, Institute of Geochemistry, Chinese Academy of Sciences, Guiyang 550081, China.; ^2^ College of Earth and Planetary Sciences, University of Chinese Academy of Sciences, Beijing 100049, China.; ^3^ Center for Excellence in Comparative Planetology, Chinese Academy of Sciences, Hefei 230026, China.; ^4^ State Key Laboratory of Ore Deposit Geochemistry, Institute of Geochemistry, Chinese Academy of Sciences, Guiyang 550081, China.

## Abstract

The existence of molecular H_2_O and evolution of solar wind–derived water on the lunar surface remain controversial. We report that large amounts of OH and molecular H_2_O related to solar wind and other multiple sources are preserved in impact glasses from Chang’e-5 (CE5) lunar soil based on reflectance infrared spectroscopy and nanoscale secondary ion mass spectrometry analyses. The estimated water content contributed by impact glasses to CE5 lunar soil was ~72 ppm, including molecular H_2_O of up to 15 to 25 ppm. Our studies revealed that impact glasses are the main carrier of molecular H_2_O in lunar soils. Moreover, water in CE5 impact glasses provides a record of complex formation processes and multiple water sources, including water derived from solar wind, deposited by water-bearing meteorites/micrometeorites, and inherited from lunar indigenous water. Our study provides a better understanding of the evolution of surficial water on airless bodies and identifies potential source and storage pathways for water in the terrestrial planets.

## Teaser

Abundant OH and molecular H_2_O derived from multiple sources are found in impact glasses from Chang’e-5 lunar soil.

## INTRODUCTION

Infrared spectrometers on Cassini, Deep Impact, and Chandrayaan-1 revealed widespread hydration on the lunar surface, and the primary source mechanism is solar wind implantation (*1*–*6*). Indigenous water from lunar interior and exogenous water delivered by water-bearing meteorites/micrometeorites and comets have also been proposed to contribute partially to the hydration on the lunar surface (*7*–*9*). Solar wind radiation damages the surface structure of lunar grains, which allows implanted protons to be retained as H and H_2_ in defects, and to combine with suspended oxygen atoms in the broken bonds to form OH and even H_2_O (*10*, *11*). All such H-bearing species derived from solar wind are referred as solar wind–derived water. The existence of molecular H_2_O on the lunar surface was not confirmed until NASA/DLR Stratospheric Observatory for Infrared Astronomy (SOFIA) performed lunar observations at 6 μm. Quantitative analyses suggest that a high content of molecular H_2_O occurs at high lunar latitudes and formed in situ from preexisting OH during micrometeorite impacts (*12*, *13*). The Apollo and Chang’e-5 (CE5) lunar sample studies provide direct measurement evidence for solar wind–derived water from lunar minerals and impact glasses (*14*–*18*). However, molecular H_2_O was only identified in one plagioclase with a high content of solar wind–derived water from CE5 lunar soils (*17*). Thus, lunar sample analyses have not confirmed the prevalence and content of molecular H_2_O on the lunar surface. Impact glass is an important component of lunar soils, and it includes pure glass particles with a homogeneous structure and composition, porous agglutinitic glasses, and amorphous coatings over particle surfaces (*19*). Impact glasses are unique products generated by meteorite/micrometeorite impacts, and they can be exposed to solar wind implantation before and after their formation; thus, they may preserve molecular H_2_O. Impact glasses can record meteorite/micrometeorite impact and solar wind implantation processes on the lunar surface, which is important for understanding the formation, species, and preservation of lunar surficial water.

Previous studies have investigated the water in impact glasses from Apollo and CE5 lunar samples, and they revealed that impact glass is an important solar wind–derived water reservoir; although the species of water is unclear (*15*, *16*, *18*). Liu *et al.* (*14*) reported that the water in Apollo agglutinitic glasses existed mainly in hydroxyl form derived from solar wind sources. However, the samples were doubly polished for analyses of interior water rather than water directly from the surface implanted by the solar wind. In addition, although investigations on CE5 lunar impact glasses have confirmed the existence of solar wind–derived water and proposed that the water in impact glass beads could act as a buffer to sustain the lunar surficial water cycle (*16*, *18*), other sources of water that may be recorded in the impact glasses have not been investigated. The lack of insights into the species and sources of water in impact glasses limits our understanding of the unique formation and preservation processes of lunar surficial water in lunar soils.

The CE5 mission returned 1731 g of lunar samples from the northeastern Oceanus Procellarum basin on 17 December 2020 (*20*), which provided an opportunity to reveal the formation and preservation processes of solar wind–derived water in impact glasses. Moreover, CE5 samples collected from the northeastern Oceanus Procellarum basin at middle latitude (43.06°N, 51.92°W), which is higher than the latitude of all previous Apollo and Luna sampling missions (*21*, *22*), could provide insights into the effects of different environment temperatures and lunar soil maturity on solar wind–derived water in impact glasses. Twelve CE5 impact glasses grains were selected to investigate the effective formation of solar wind–derived water in impact glasses and the influence of meteorite/micrometeorite impacts and solar wind implantation on the formation and preservation of lunar surficial water. Reflectance infrared (IR) spectra of these grains were obtained to analyze the species and contents of water in the CE5 lunar glasses. Nanoscale secondary ion mass spectrometry (NanoSIMS) was performed to investigate the distribution of hydrogen isotope compositions and hydrogen contents from the uppermost surface to the interior (~1400 nm depth) of the lunar glass grains. Scanning electron microscopy (SEM) and electron probe microanalyzer (EPMA) investigations were performed to determine the structure and composition of the glasses. By investigating the water in lunar impact glasses, the preservation and species of water were determined and multiple sources of water were identified, which confirmed the important contribution of impact glasses to lunar surficial water. This study can help understand the distinctive formation and preservation of water in impact glasses via space weathering processes on the lunar surface.

## RESULTS AND DISCUSSION

### Water abundance and origin of CE5 lunar impact glasses

**Fig. 1.**
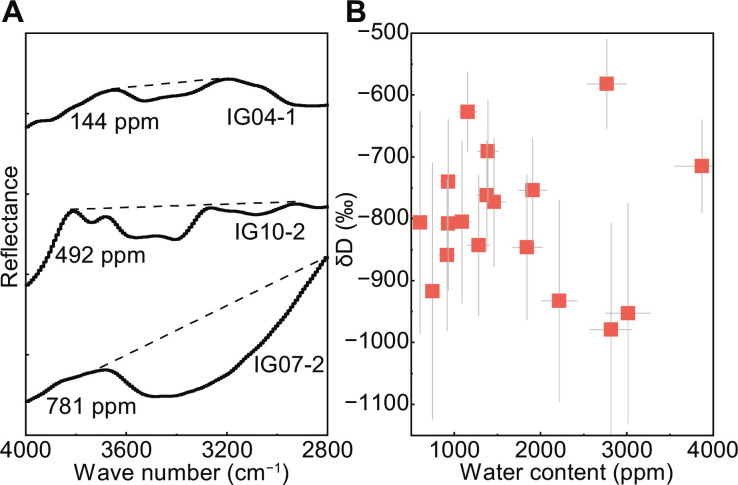
Water content and hydrogen isotope composition in CE5 impact glasses. (**A**) Representative reflectance infrared (IR) spectra in the range of 2800 to 4000 cm^−1^ for CE5 impact glasses. The solid lines are the smoothed spectra obtained using the fast Fourier transform algorithm, and the dashed lines are the baseline positions for the OH/H_2_O content estimations. (**B**) Water content and hydrogen isotope composition (δD) within the uppermost ~1400 nm of CE5 impact glasses determined via nanoscale secondary ion mass spectrometry (NanoSIMS). δD = (D/H_sample_/D/H_VSMOW_) − 1 × 1000; VSMOW represents the Vienna standard mean ocean water. The error bars (gray line) represent 2σ, which corresponds to the uncertainty of the reproducibility of D/H measurements on the standards, uncertainty of H_2_O background subtraction, internal precision, and uncertainty of the depth measurements.

A total of 12 impact glasses were selected from CE5 lunar soils for detailed investigation, including agglutinates, pure glasses, and amorphous coatings (fig. S1). Reflectance IR spectra of 11 CE5 impact glasses show broad absorption in the range of 2800 to 4000 cm^−1^, with bands centered around 3400 to 3550 cm^−1^ (Fig. 1A). This indicates the presence of OH groups in glasses in the form of OH and/or H_2_O (denoted by OH/H_2_O) (*23*). Using a calibration line from terrestrial glasses, we calculated the bulk OH/H_2_O content in the impact glasses by the depth of the absorption band (see Materials and Methods and fig. S2). The results reveal a high abundance of OH/H_2_O in the CE5 impact glasses compared to that in lunar interior (*24*, *25*), with water content ranging from 144 ± 36 to 781 ± 193 parts per million (ppm) (435 ± 107 ppm average) (table S1 and data S1). The NanoSIMS analyses indicated that the equivalent water content (including all the H-bearing species) within approximately 1400 nm of the CE5 impact glasses was 602 ± 64 to 3874 ± 324 ppm (Fig. 1B and table S2). The water content obtained by the reflectance IR spectra is generally lower than that obtained by the NanoSIMS measurements, suggesting that water is concentrated in the uppermost layer. This is the typical distribution of solar wind–derived water within the grains (*16*, *26*). After correcting for the effects of cosmic ray spallation (see Materials and Methods) (*25*, *27*), the hydrogen isotope composition (expressed as δD) within the 1400 nm depth of glasses surface mainly ranged from −979 ± 172 to −582 ± 72 per mil (‰; see Materials and Methods and Fig. 1B and table S2). The low δD provides further evidence that the water in impact glasses is mainly derived from solar wind, which has a δD value of approximately −1000‰ (*28*).

Our NanoSIMS results for the water content in CE5 impact glasses are consistent with the findings of previous studies on lunar impact glasses from Apollo samples determined by NanoSIMS (*15*), whereas the reflectance IR spectra results are higher than those determined by Fourier transform IR spectroscopy (70 to 170 ppm), which measured the water content inside the samples after double-polishing the grains (*14*). Solar wind–derived water is mainly retained in the uppermost ~200 nm of lunar grains (*17*); thus, the water content would be underestimated after the double-polish treatment. On the basis of the bulk OH/H_2_O content determined from the reflectance IR spectra of the impact glasses and the average content of glasses in the CE5 lunar soils (~16.6%) (*21*), we estimated that the water content of the impact glasses contributed to CE5 lunar soils was ~72 ppm. Combined with the water content in CE5 minerals (*17*), we conclude that the water abundance of lunar soils in the CE5 region is approximately 242 ppm. Moreover, the water in CE5 lunar soils occurs mostly as OH/H_2_O and originates mainly from solar wind (with δD close to −1000‰) (*28*).

### Molecular H_2_O in CE5 impact glasses

**Fig. 2.**
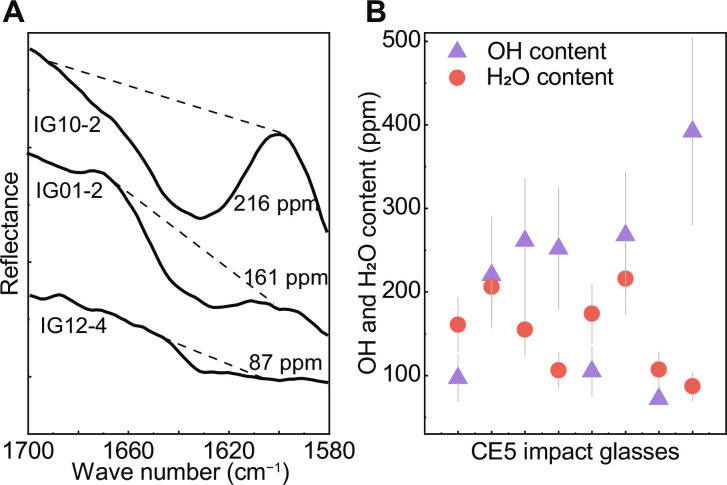
Molecular H_2_O content in CE5 impact glasses. (**A**) Representative reflectance IR spectra in the range of 1580 to 1700 cm^−1^ for CE5 impact glasses. The solid lines indicate the smoothed spectra obtained using the fast Fourier transform algorithm. The dashed lines indicate the baseline of H_2_O absorption. (**B**) Comparison of the OH content (i.e., bulk OH/H_2_O content minus the bulk molecular H_2_O content) and molecular H_2_O derived from reflectance IR spectra of CE5 impact glasses. The triangles and circles represent the bulk OH content and bulk molecular H_2_O content, respectively. The gray lines are the error bars. The uncertainty of the estimated OH content includes errors in measurements conducted for terrestrial glasses, counting statistics of each analysis, and reflectance IR measurements. The uncertainty of the calculated molecular H_2_O content is ~20% (*4*).

A distinct absorption band centered around 1630 cm^−1^ was observed in seven impact glasses, which is ascribed to molecular H_2_O (Fig. 2A) (*29*). H_2_O absorption likely did not occur by atmospheric water because these samples were not exposed to terrestrial air and had very low δD in the surface. Measurements of nominally anhydrous mineral references showed no atmospheric water interference during our reflectance IR measurements (see Materials and Methods and fig. S3). The absorption was also not due to the presence of Mg-SiO, as there was no observable absorption near 2000 cm^−1^ (fig. S4) (*30*). Thus, we concluded that the molecular H_2_O measured by reflectance IR is present within the impact glasses. According to the radiative transfer theory of Milliken and Li (*8*) with grain size and the extinction coefficient of silicate glasses at 6.1 μm (1630 cm^−1^), the estimated H_2_O content in CE5 impact glasses was 87 ± 17 to 216 ± 42 ppm (see Materials and Methods and table S3). The molecular H_2_O content accounts for 0.18 to 0.61 of the total water content calculated at a wave number of 2800 to 3800 cm^−1^ (H_2_O / (OH + H_2_O) ratio; Fig. 2B). According to the content of molecular H_2_O in the impact glasses, the molecular H_2_O content in CE5 lunar soils was estimated to be 15 ± 3 to 25 ± 5 ppm (see Materials and Methods). This value is lower than the approximately 100 to 400 ppm estimated by Honniball *et al.* (*12*) using the spectral signature at 6 μm obtained from the NASA/DLR SOFIA. On the basis of the study of the species of water in CE5 lunar minerals reported by Zhou *et al.* (*17*), we concluded that molecular H_2_O is mainly retained in the lunar impact glasses. This is consistent with the view that molecular H_2_O can be more effectively preserved in glass at high latitudes (*12*). The low content of molecular H_2_O in CE5 lunar soils is probably a result of the lower latitude of the CE5 sampling site (43.06°N) compared with that of the research area (55.6°S-75°S) studied by Honniball *et al.* (*12*) and the relatively low content of impact glasses in CE5 lunar soils.

Three potential mechanisms may account for the formation of molecular H_2_O in impact glasses. First, molecular H_2_O is formed from the direct implantation of solar wind protons into impact glasses, although this mechanism is still controversial (*10*, *11*, *31*). Second, impact glasses preserve molecular H_2_O through the impact delivery of H_2_O-rich meteorites and micrometeorites (*9*). Third, these impacts induced the formation of molecular H_2_O from solar wind–derived OH contained in lunar soil before the impact event. Simulation experiments have reported that ion implantation followed by micrometeorite impact can effectively produce and release molecular H_2_O (*32*). It is possible that rapid quenching may preserve part of the molecular H_2_O in the subsequently produced impact glasses (*9*). The latter is the most likely mechanism for molecular H_2_O formation, considering that previous studies on lunar minerals have not found widespread H_2_O (*17*) and that the δD value in the interior of some impact glasses is close to that of solar wind (analyzed below) (*14*).

### Indication of multiple sources of water preserved in CE5 lunar impact glasses

**Fig. 3.**
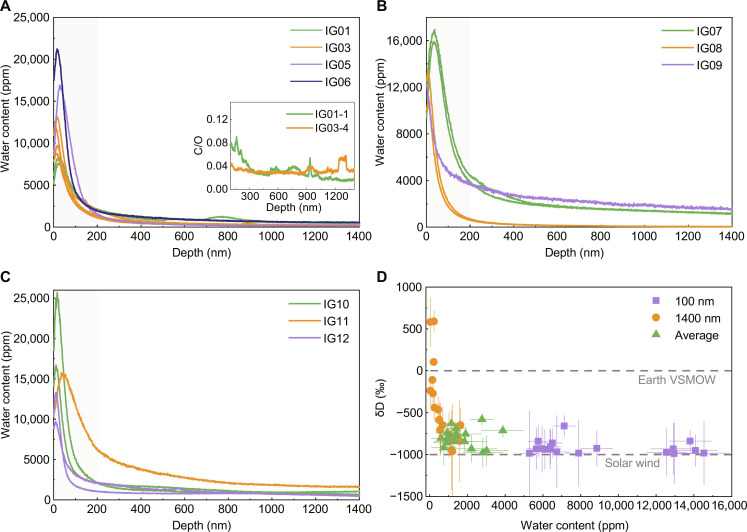
Water content and hydrogen isotope composition distribution in CE5 impact glasses. (**A** to **C**) Depth profiles of water content in CE5: (A) agglutinates, (B) pure glasses, and (C) amorphous coatings measured by NanoSIMS. The gray areas show the typical distribution of solar wind–derived water with a rapid increase in water content followed by a rapid decrease. (**D**) Water content and δD at different depths of the CE5 glasses. The squares and circles represent the average water content and δD within the first 100 nm (surface) and to a depth of 1300 to 1400 nm (interior), respectively. The triangles represent the average water content and δD in the depth of 1400 nm. The error bars represent 2σ.

To investigate the distribution and possible multiple sources of water in CE5 impact glasses, the profile characteristics of hydrogen abundance and hydrogen isotope composition from the uppermost surface to the interior (~1400 nm) were analyzed by NanoSIMS. The profile of the CE5 impact glasses showed a similar distribution of water content (equivalent to H abundance) and hydrogen isotope composition (Fig. 3). The water content in the uppermost surface (<50 nm) initially increased sharply to a maximum amount of 7625 to 25919 ppm. Subsequently, the water content decreased rapidly to a depth of approximately 200 nm and then began to decrease slowly (Fig. 3, A to C). The uppermost ~100 nm presents highly D-poor water, with a δD value of −988 ± 364 to −659 ± 124‰, and most values were lower than −840‰, revealing that all the impact glasses were subject to solar wind implantation followed by diffusion after formation (Fig. 3D). However, wide ranges of δD (−962 ± 556 to 590 ± 129‰) and water content (37 ± 3 to 1628 ± 131 ppm) were observed at a depth of 1300 to 1400 nm (table S2). Considering that the solar wind is highly D-poor, the diffusion of solar wind–derived water leads to limited hydrogen isotope fractionation; thus, the interior water cannot be contributed solely by the diffusion of solar wind–derived water. The EPMA results showed that the abundance and δD of water were not notably correlated with the type and composition of impact glasses (table S4). Thus, the water in CE5 impact glasses is likely characterized by complex impact processes and multiple sources.

**Fig. 4.**
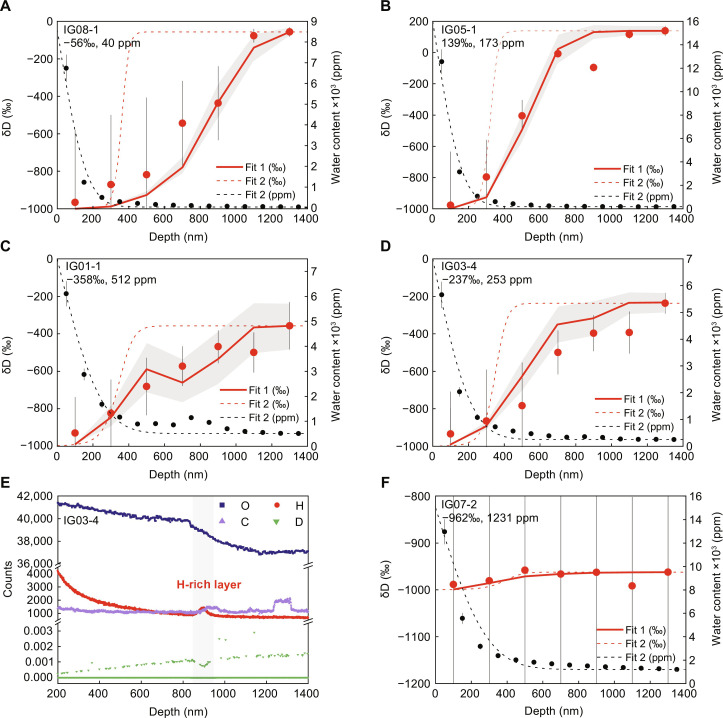
Multiple sources of water recorded in CE5 impact glasses. (**A** to **D**) Fitting results for the distribution of water content and δD in CE5 impact glasses: (A) IG08-1, (B) IG05-1, (C) IG01-1, and (D) IG03-4. The red and black circles represent the δD values and water content at different depths as measured by NanoSIMS. The δD and content of water at a depth of 1200 to 1400 nm (labeled in upper left) were taken as the δD and content of interior water in impact glass, respectively. The δD distribution in impact glasses are well reproduced by a binary mixing model (red solid lines), which assumes that the δD distribution in impact glasses is the result of mixing of interior water with the solar wind–derived water. The shaded areas are the errors of the fit for the binary mixing models calculated from the errors of water content and δD. The black dashed lines are the fitting results of water content based on the diffusion model; whereas the red dashed lines are the fitting results of δD based on modeling of water content based on the diffusion model. More details of modeling are described in the Materials and Methods. The difference between the solid and dashed red lines indicates that the implantation-diffusion process in impact glasses was multistaged, rather than a continuous implantation-diffusion process. All error bars (gray line) correspond 2σ. (**E**) Distribution of oxygen (O), hydrogen (H), carbon (C) counts, and D/H ratios at depths of 200 to 1400 nm for the grain IG03-4. The gray area corresponds to the H-rich layer at a depth of 850 to 950 nm. (**F**) Fitting results for the distribution of water content and δD in impact glass IG07-2. The symbols and lines are consistent with those in (A) to (D).

Three potential sources contributed to the water in lunar soils: implantation by solar wind protons, which has a δD close to −1000‰ (*17*, *18*); indigenous water from lunar interior volcanic degassing, which has a δD from −330 to 1000‰ (*25*, *33*, *34*); and exogenous water delivered by carbonaceous chondrites, which have a δD of approximately −400 to 100‰ (*35*), and by comets, which have a δD of approximately −100 to 2500‰ (*36*, *37*). On the basis of a binary mixing model, which assumed the δD profiles in impact glasses resulted from the mixing of solar wind implantation at a depth of 0 to 200 nm and interior water at a depth of 1200 to 1400 nm (see Materials and Methods), the multiple sources of water recorded in CE5 impact glasses are discussed. The binary mixing model well reproduces the five δD profiles, indicating the mixture of solar wind–derived water and interior water in the impact glasses (Fig. 4). Other δD profiles are very difficult to fit, suggesting intricate thermal and kinetic history and/or multiple sources of water (figs. S5 and S6). In addition, the interior water may be produced by degassing of the impact glass precursors during impact process. However, all the δD of the lunar volcanic glasses and melt inclusions can be reproduced by a degassing model with an empirical fractionation factor β ≤ 0.06, and the largest fractionation by degassing is <250‰ (*24*). Therefore, the δD of interior water still indicates the source of water, as discussed in the following section.

The content and δD of the interior water in CE5 impact glasses and the fitting results indicate that multiple sources of water can be preserved in impact glasses. For example, the analysis of grains IG08-1 and IG05-1 suggested a mixed source of highly D-poor solar wind–derived water and interior water with δD of −56 and 139‰, respectively (Fig. 4, A and B). Combined with the low interior water content (40 ppm in IG08-1 and 173 ppm in IG05-1), it is likely that these impact glasses preserved the lunar indigenous water. This indicates that indigenous water was lost by degassing in the form of H_2_, and minor amounts were preserved during the formation of the impact glasses, after which the implantation of solar wind protons induced high water contents on the surface.

The water content and δD of IG01-1 and IG03-4 represent a mixture of a solar wind source and a water-rich but relatively D-poor source (Fig. 4, C and D). Impact process may cause the loss of water with lighter isotopes in the precursors, the δD and water content in the interior of IG01-1 and IG03-4 represent the maximum δD (−358 and −237‰) and minimum water content (512 and 253 ppm) in their precursors, respectively. Previous studies on the CE5 basalts have yielded a maximum water content of 283 ppm and a δD of −330‰ for the parent magma of CE5 samples (*25*). Although apatite grains in CE5 samples contain high water abundances (550 to 4856 ppm), apatite was the last crystallization product of magma degassing, and it had a high hydrogen isotope composition with δD > 275‰ (*25*). Thus, the relatively low δD and high content of water in the interior of impact glass grains IG01-1 and IG03-4 indicate that the water-rich carbonaceous chondrite contributed at least some of the water to the impact glasses. In addition, the carbon/oxygen ratios of IG01-1 and IG03-4 determined by NanoSIMS showed several anomalous elevations in the interior (Fig. 3A), further supporting the addition of water of carbonaceous chondrites during the impact event.

An H-rich layer was found in the NanoSIMS profile of grain IG03-4 at a depth of 850 to 950 nm (Fig. 4E). The distribution of oxygen and carbon in grain IG03-4 showed a break at a depth of 850 nm. At depths greater than 850 nm, the abundance of carbon increased notably and oxygen decreased; moreover, the lower D/H ratios in the H-rich layer with 100 nm thickness showed a similar H-abundance profile to solar wind protons implanted in grains. These findings imply that the precursor grain underwent the incorporation of a carbonaceous chondrite–like impactor and then was exposed to space and implanted by solar wind protons. Subsequently, ejected melt from elsewhere covered the surface of the grain, causing the solar wind–derived water in the H-rich layer to partially escape under the thermal effects.

In addition, the water in the interior of impact glass IG07-2 still had high content of 1231 ppm and low δD of −962‰ (Fig. 4F). Such extremely D-poor water indicates the contribution of solar wind implantation. Grain IG07-2 exhibited a homogeneous structure with low FeO and TiO_2_ and high Al_2_O_3_ and SiO_2_ (table S4), indicating formation based on the rapid cooling of exotic components at the CE5 region, most likely from the Aristarchus crater (*22*, *38*). Considering the water content, hydrogen isotope composition characteristics, and large impact event, it is possible that the precursor of IG07-2 contained abundant solar wind–derived water, part of which was retained in impact glass IG07-2 after the impact process and then the solar wind implanted hydrogen into IG07-2 to form the observed water. The depth distribution of water in IG03-4 and IG07-2 indicates that ancient solar wind–derived water can be preserved in impact glasses.

The profile distribution of the water and hydrogen isotope composition in impact glasses indicates that the CE5 lunar impact glasses generally contained abundant water formed by solar wind implantation. The impact glasses can record different sources of water retained during different impacts, including ancient solar wind–derived water, lunar indigenous water, and water delivered by meteorites (micrometeorites) or comets. The water from the impactors and impact targets that can be retained in the lunar impact glasses is consistent with the conclusion of the impact experiment by Daly and Schultz (*9*), which confirmed that the impact-produced melt hosts the bulk of the delivered water. The water in the impact glasses provides important information on the impact processes experienced by lunar grains and has important implication for investigating the preservation of water on the lunar surface.

### Implications for the formation and evolution of water in the solar system

Impact glasses, as important components of lunar soils, have higher water contents than minerals because of their unique structure and formation history (*17*). The water content contributed by impact glasses to the CE5 lunar soils is approximately 72 ppm. Our study reveals that molecular H_2_O can be retained in lunar impact glasses, and it was likely formed through meteorite/micrometeorite impacts and melting of solar wind–rich soils; however, this mechanism requires further investigation. Given that the abundance of impact glass increases with the degree of space weathering (i.e., maturity) of the lunar soils (*39*) and the relative immaturity of CE5 lunar soil (*40*, *41*), the contribution of impact glasses to lunar soil water and the abundance of molecular H_2_O in lunar impact glasses should be greater in a mature soil. Our studies reveal that impact glass is an important reservoir for lunar surficial water, in particular for the preservation of molecular H_2_O.

This study has important implications for the investigation of water ice in polar regions. In the illuminated area, the exposure of lunar soil to solar wind implantation followed by meteorite/micrometeorite impacts may cause a portion of the released molecular H_2_O to migrate and accumulate in cold traps in the polar region (*42*). Water ice was detected in the polar region using the Moon Mineralogy Mapper (M3) instrument, and a general shift in absorption from 1.5 μm (the absorption of pure water ice) toward 1.55 μm was found in the M3 spectra (*43*). Although Li *et al.* (*43*) proposed that such band shifts may reflect low-density ice in lunar soils, our study provides an alternative possibility that molecular H_2_O in the lunar polar regions may be preserved in agglutinates, which can also cause the same spectral shifts (*44*). In the lunar polar region, molecular H_2_O can be trapped between grains in permanently shadowed regions and stabilized via sequestration in impact glasses. This finding provides insights into approaches to determine the sources and preservation of water in the lunar polar region.

Solar wind implantation and meteorite/micrometeorite impacts are also prevalent on the surfaces of other airless terrestrial bodies, such as Mercury and asteroids, and the molecular H_2_O and multiple sources of water are expected to be preserved in the impact glasses of regolith, which would have an important impact on the formation and evolution of water on the surface of these bodies. Similarly, in solar nebula and debris disk periods, water can be produced in micrometer-size dust particles by solar wind proton implantation and/or hydrogen diffusion (*45*). With the collision of these dust particles to form planetesimals, part of the water could be fixed in impact melts and eventually transported to the interior of the terrestrial planets through the accretion and growth process of planetesimals (*9*). This represents a feasible source and storage pathway for water in the early interior of terrestrial planets.

## MATERIALS AND METHODS

### Sample collection and preparation

Lunar glass grains used in this study were selected from CE5 lunar soil (CE5C1000YJFM00303, ~500 mg), which was shoveled from the lunar regolith. The CE5 lunar samples were stored in an ultraclean room at Extraterrestrial Sample Curation Center of National Astronomical Observatories, Chinese Academy of Sciences (CAS), before being allocated by the China National Space Administration. Dozens of lunar grains, with grain sizes ranging from 100 to 300 μm and displaying distinctive characteristics of impact glasses (i.e., with the glassy luster, black or brown in color), were handpicked under binoculars in a glovebox with flowing high-purity argon at the Institute of Geochemistry, CAS. All samples were unpolished, and exposure to terrestrial air was prevented as much as possible before the reflectance IR and NanoSIMS measurements. Sample transfer was completed with the grains in sealed boxes filled with high-purity argon to prevent the samples from being exposed to terrestrial air. After reflectance IR and NanoSIMS measurements, the samples were observed by SEM and then polished for EPMA analyses. The types of CE5 samples were determined based on the reflectance IR spectra in the 650 to 1300 cm^−1^ range (fig. S7), microstructural characteristics measured using SEM (fig. S1), and chemical composition measured using EPMA (table S4). Twelve of CE5 impact glasses were then selected for a detailed discussion of the characteristics and sources of water.

### Reflectance IR spectra measurements

Reflectance IR spectra of the samples were obtained with a Nicolet iS50 FTIR Spectrometer coupled with a Continuum IR microscope (Thermo Fisher Scientific) at the Institute of Geochemistry, CAS. The IR microscope was installed in a glovebox and continuously purged by high-purity nitrogen throughout all the measurements. In the high-purity argon glovebox, all selected grains were placed on cooper meshes embedded in a customized ceramic plate and then placed into a sealed box. Then, the samples were transferred to the glovebox of the IR spectrometer. The reflectance IR spectra of untreated lunar grains were collected in the range of 650 to 8000 cm^−1^. Each spectrum was scanned 256 times with a 4 cm^−1^ resolution, and the aperture size was set to 75 μm × 75 μm. Background correction of the spectra was performed using a gold standard. Before and during the reflectance IR measurements, the lunar grains were not exposed to terrestrial air; therefore, any possible interference from terrestrial air can be eliminated. The lunar grain phases were determined by the reflectance IR spectra, and impact glasses were then selected for further investigation.

The OH/H_2_O content of lunar impact glasses was calculated based on the calibration line derived from the analyses of terrestrial volcanic glasses, which was obtained by linearly fitting the OH/H_2_O absorption depth in the range of 2800 to 3800 cm^−1^ as measured by Fourier transform IR (FTIR) and water content as measured by NanoSIMS (fig. S2). The OH/H_2_O absorption depth was determined by measuring the distance from the lowest point of OH absorption around 3550 cm^−1^ to the baseline, which was determined from the two highest points adjacent to the absorption band. The terrestrial glasses are natural low-Ti basalt glasses from the Changbai Mountains of northeast China and are the primary material used in the preparation of the CAS-1 lunar soil simulant (*46*). First, the reflectance IR spectra of unpolished terrestrial glass grains were collected using the same methods as for the lunar impact glasses. The depth of absorption around 3550 cm^−1^ in the IR spectra was measured. Subsequently, the water content of the unpolished terrestrial glass grains was determined by NanoSIMS at the Institute of Geology and Geophysics, CAS. The spots selected for the NanoSIMS analyses coincided with the areas of the reflection IR spectra measurements. On the basis of the reflection IR and NanoSIMS measurements in the same area of each grain, the relationship between depth of absorption around 3550 cm^−1^ and OH/H_2_O was determined (table S5). The slope of the calibration is (1.87 ± 0.08) × 10^−4^. All OH/H_2_O content results are reported with 2σ uncertainties, including the error in counting statistics of each analysis from NanoSIMS (~1.84%), the error in calibration line (~4.28%), and the uncertainty in absorption band depth from FTIR (~20%).

### Atmospheric interference exclusion and molecular H_2_O content calculation

The molecular H_2_O content was obtained from the reflectance of the absorption band centered around 1630 cm^−1^. Since the CE5 impact glasses were not exposed to terrestrial air before the FTIR measurements and were continuously purged by high-purity nitrogen during spectra collection, any interference from atmospheric water can be excluded. In addition, same measurements were conducted on San Carlos olivine references, which are nominally anhydrous minerals. The reflectance IR spectra of San Carlos olivine showed no absorption for either OH or H_2_O, thus confirming the lack of atmospheric water interference in our measurements (fig. S3).

To calculate the molecular H_2_O content in CE5 impact glasses, the correlation between the reflectance at 1630 cm^−1^ and H_2_O content was determined using the radiative transfer theory and the Beer-Lambert law (*47*, *48*), which has also been applied by Milliken and Li (*8*) and Honniball *et al.* (*12*). The spectra were measured on the aperture area, which is smaller than all the measured surface areas. In addition, the reflectance was treated as single scattering albedo of single grains. The continuum was removed to isolate the absorption of molecular H_2_O before calculation (*49*, *50*). The continuum removal was performed over the 1700 to 1580 cm^−1^ wave number range, which covers the absorption band of molecular H_2_O for all CE5 impact glasses. Afterward, the molecular H_2_O content was calculated based on the equivalent slab model and the Beer-Lambert law for each glass grain. The extinction coefficient at 1630 cm^−1^ used in our calculation was 42.34 l mol^−1^ cm^−1^ (*29*), and other parameters were similar to those used in Milliken and Li (*8*). The reflectance and grain size of the impact glasses are listed in table S3. The uncertainties of molecular H_2_O content calculated from reflectance IR spectra were ~20%. The presence of other absorptions near 1630 cm^−1^ in some impact glasses may overlap with or mask the absorption of molecular H_2_O. Assuming that all impact glasses contained H_2_O, the estimated H_2_O content in CE5 lunar soils was 25 ± 5 ppm based on the average H_2_O content in impact glasses and the glass abundance in CE5 lunar soils. In addition, if H_2_O exists only in impact glasses with identifiable absorption at 1630 cm^−1^ in the reflectance IR spectra, then the H_2_O content in CE5 lunar soils would be 15 ± 3 ppm, which represented the lower limit.

### NanoSIMS analyses

NanoSIMS was used to obtain both the hydrogen isotope composition and hydrogen content on the profiles of the CE5 impact glasses from the uppermost surface to the interior (~1400 nm). After the FTIR measurements, the samples were sealed and transferred to the high-purity argon glovebox. The impact glasses determined by reflectance IR spectra were selected and embedded in indium discs. The sample surfaces were then coated with Au to prevent charging during the NanoSIMS analyses and loaded in sample holders with a set of standards; the process had an exposure time of less than 10 min. The hydrogen isotopes and water content of the impact glasses were measured using a CAMECA NanoSIMS 50L at the Institute of Geology and Geophysics, CAS, as reported by Hu *et al.* (*51*, *52*). Both the samples and standards were loaded in sample holders together and were kept in the NanoSIMS airlock for more than 24 hours. Then, the holders were stored in NanoSIMS analysis chamber until the vacuum pressure was sufficiently low (2.8 × 10^−10^ to 3.0 × 10^−10^ mbar) to minimize the hydrogen background. Each 10 μm × 10 μm relatively flat area without cracks was identified (as viewed by the real-time imaging mode of NanoSIMS) and presputtered for ~10 s with a Cs^+^ ion beam current of ~50 nA, and analyses were performed using a Cs^+^ ion beam with a beam current of 300 pA on the central 5 μm × 5 μm area using the blanking technique. The secondary anions ^1^H^−^, ^2^D^−^, ^12^C^−^, and ^18^O^−^ were simultaneously collected on electron multipliers for 1500 cycles. The depths of the NanoSIMS measurement spots were obtained by measuring the cross section of the two spots prepared by the focused ion beam. The results showed that the depth of each NanoSIMS measurement cycle was ~0.95 ± 0.8 nm (fig. S8). Combined with the depth of NanoSIMS spots on CE5 impact glasses measured by Xu *et al.* (*16*), the error for the depth of NanoSIMS spots was estimated to be ~8%. A 44 ns dead time was corrected for all EMs, and the charging effect on the sample surface was compensated by an electron gun during the analyses.

The water content of the CE5 impact glasses was calculated from the ^1^H^−^/^18^O^−^ ratio multiplied by the slope of the calibration base on a set of terrestrial standards for which the water content has been reported, including Kovdor apatite, basaltic glass 1838, SWIFT MORB glass, and Durango apatite (*25*). The slope of the calibration line was (7.15 ± 0.06) × 10^−5^ (fig. S9). The H_2_O background corrections of the instrument were performed using the standards of anhydrous San Carlos, whose water content was determined to be 12 ± 2.07 ppm. This value was subtracted from each of the reported water content values of the CE5 impact glasses. Notably, the water content measured by NanoSIMS includes the content of all the H-bearing species, such as H, H_2_, OH, and H_2_O. The correction of instrumental mass fractionation (IMF) on hydrogen isotope composition was conducted using the Kovdor apatite standard. All reported values of hydrogen isotope composition were corrected for the IMF, and the average IMF factor was calculated to be 1.05 ± 0.025. The hydrogen isotope composition of CE5 impact glasses was expressed using the delta notation, δD = {[(D/H)_sample_/(D/H)_VSMOW_] − 1} × 1000‰, where D/H_VSMOW_ = 1.56 × 10^−4^ (*7*). The δD were then corrected for the effects of cosmic-ray spallation using the D production rate of 2.17 × 10^−12^ mol D g^−1^ Ma^−1^ (*27*). Correction for cosmic-ray spallation on water content was not conducted as the effect is negligible (*24*). The cosmic-ray exposure age used for correction was 50 Ma, which was estimated by Hu *et al.* (*25*). In our study, all water content and δD values were reported with 2σ uncertainties, including the reproducibility of D/H measurements on the standards, uncertainty of H_2_O background subtraction, internal precision, and uncertainty of the depth measurements. In addition, all reported water content and δD values within the different depths represented the average values within the corresponding depths.

### SEM analyses

To confirm the position of the NanoSIMS analyses areas and identify the types of CE5 impact glasses, surface morphology observations and composition analyses of the impact glasses were carried out using an FEI Scios dual-beam SEM equipped with an energy-dispersive x-ray spectrometry at the Institute of Geochemistry, CAS. The acceleration voltage was 15 to 25 kV and the electron beam current was 0.8 to 3.2 nA. Subsequently, the representative impact glasses were extracted from the indium discs and mounted in epoxy in the same orientation as the above measurements to prepare the polished sections. These sections were observed via SEM again to further determine the interior structure and types of impact glasses. Back-scattered electron images of the initial and polished CE5 impact glasses are shown in fig. S1.

### EPMA measurements

To obtain the composition of CE5 impact glasses, the lunar grains were dug out from indium discs using a high-precision tweezers and placed on double-sided tape under binoculars. Then, these grains on the tape were mounted in epoxy and polished sections were prepared manually using sandpaper and alumina powder under binoculars. Before EPMA measurements, the polished grains were cleaned with ethanol and coated with carbon. Quantitative composition analyses of CE5 impact glasses polished sections were performed using a JXA 8230 EPMA at the Institute of Geochemistry, CAS (table S4). The operating accelerating voltage was 30 kV, the beam current was 10 nA, and the beam size was 1 to 5 μm. Natural glasses and synthetic glasses were used as standards. The detection limits for most of the analytical elements were 0.01 to 0.03 wt% (*53*).

### Binary mixing modeling

To investigate the water sources in CE5 impact glasses, a binary mixing model was used for fitting. To minimize the systematic error, the water content and hydrogen isotope composition within the ~1400 nm depth were separated into 14 and 7 layers, with layer thicknesses of 100 and 200 nm, respectively. Homogenous initial water contents and hydrogen isotopic compositions are set in the glass before solar wind implantation because any heterogeneity or difference in water and hydrogen isotope compositions caused by degassing and/or diffusion processes is quite low between two points with a 1400 nm distance interval (*18*, *54*).

The error function-like water compositional profiles, supporting a diffusion-limited mechanism, can be described using the following equation

$$C=C_{\infty }+\left(C_{0}-C_{\infty }\right)\text{erfc}\frac{x}{2\sqrt{\mathrm{Dt}}}$$ (1)

where *C*, *C*_0_, and *C*_∞_ are fitting, interfacial, and initial water content. *x* is distance from the rim to the center of the glass, and *D* and *t* are diffusion coefficient and time, respectively. Erfc is the complimentary error function. Although the diffusion coefficients of solar wind hydrogen, which are functions of temperature, have been obtained based on heating experiments (*16*), the temperature or thermal history of the investigated glassy beads are not known; therefore, we treat *Dt* in Eq. 1 as a single variable. To fit the observed water content profiles in the impact glasses, the two free parameters, *C*_0_ and *Dt*, can be obtained by inversion using a penalty function approach. The selected penalty function for minimization is as follows

$$g\left(C_{0},\mathrm{Dt}\right)=\sum_{i=1}^{n}{\left(C_{i,H_{2}O}^{\text{fit}}-C_{i,H_{2}O}^{\text{obs}}\right)}^{2}$$ (2)

where *g* is the penalty function, and *i* refers to the position from the rim to the center of the glass. $C_{i,H_{2}O}^{\text{fit}}$ and $C_{i,H_{2}O}^{\text{obs}}$ are fitting and analytical data of water at position *i*, respectively. The initial water content, *C*_∞_, is set as the analytical water data at a depth of 1400 nm. The obtained *C*_0_, *Dt*, and fitting profile are illustrated in Fig. 4.

If the initially constant water content has a homogenous hydrogen isotopic composition, set as the analytical hydrogen isotope data at the depth of 1200 to 1400 nm, the isotopic composition of the implanted hydrogen is −1000‰ (δD_s_), and ignoring the isotope fractionation caused by diffusion, the isotopic composition of the mixture can be obtained as follows

$$\delta D=\frac{f_{0}C_{\infty }\delta D_{0}+f_{S}C_{S}\delta D_{S}}{f_{0}C_{\infty }+f_{S}C_{S}}$$ (3)

where *f*_0_ and *f*_s_ are the mass fraction of interior and implanted water mass, respectively. *C*_∞_ is the interior water content in the impact glass, which is the same in Eq. 1. δD_0_ is the interior hydrogen isotope composition, set as the analytical hydrogen isotope data at the depth of 1200 to 400 nm; *C*_s_ is the water content contributed by solar wind implantation. Assuming that *C*_∞_ and δD_0_ are evenly distributed initially, then δD can be obtained using Eq. 3 by assuming that the initial interior water mixed simply with the solar wind–derived water.

The fitting results for hydrogen isotopes shown in Fig. 4 are good using analytical water content data (red solid lines); conversely, using modeling results based on Eqs. 1 and 2 are misfits (reds dashed lines), indicating that modeling results for hydrogen isotopes are very sensitive to the accurate water content by solar wind implantation, because interior water content is much lower compared with the water by solar wind implantation. Some analytical compositional profiles shown in figs. S5 and S6 are very complex and challenging to fit, suggesting intricate thermal and kinetic history and/or multiple water sources.
