# Supplementary material for: Multiple sources of water preserved in impact glasses from Chang’e-5 lunar soil
Source: Sci Adv. 2024 May 10;10(19):eadl2413. doi: 10.1126/sciadv.adl2413 (PMC11086615; doi:10.1126/sciadv.adl2413)
Supplement: Supplementary file 1 — Figs. S1 to S9 Tables S1 to S5 Legend for data S1 [file sciadv.adl2413_sm.pdf]

Science Advances
AAAS

Supplementary Materials for

Multiple sources of water preserved in impact glasses from Chang'e-5 lunar soil

Chuanjiao Zhou *et al.*

Corresponding author: Hong Tang, tanghong@vip.gyig.ac.cn; Dan Zhu, zhudan@mail.gyig.ac.cn

Sci. Adv. **10**, eadl2413 (2024)
DOI: 10.1126/sciadv.adl2413

The PDF file includes:

Figs. S1 to S9
Tables S1 to S5
Legend for data S1

Other Supplementary Material for this manuscript includes the following:

Data S1

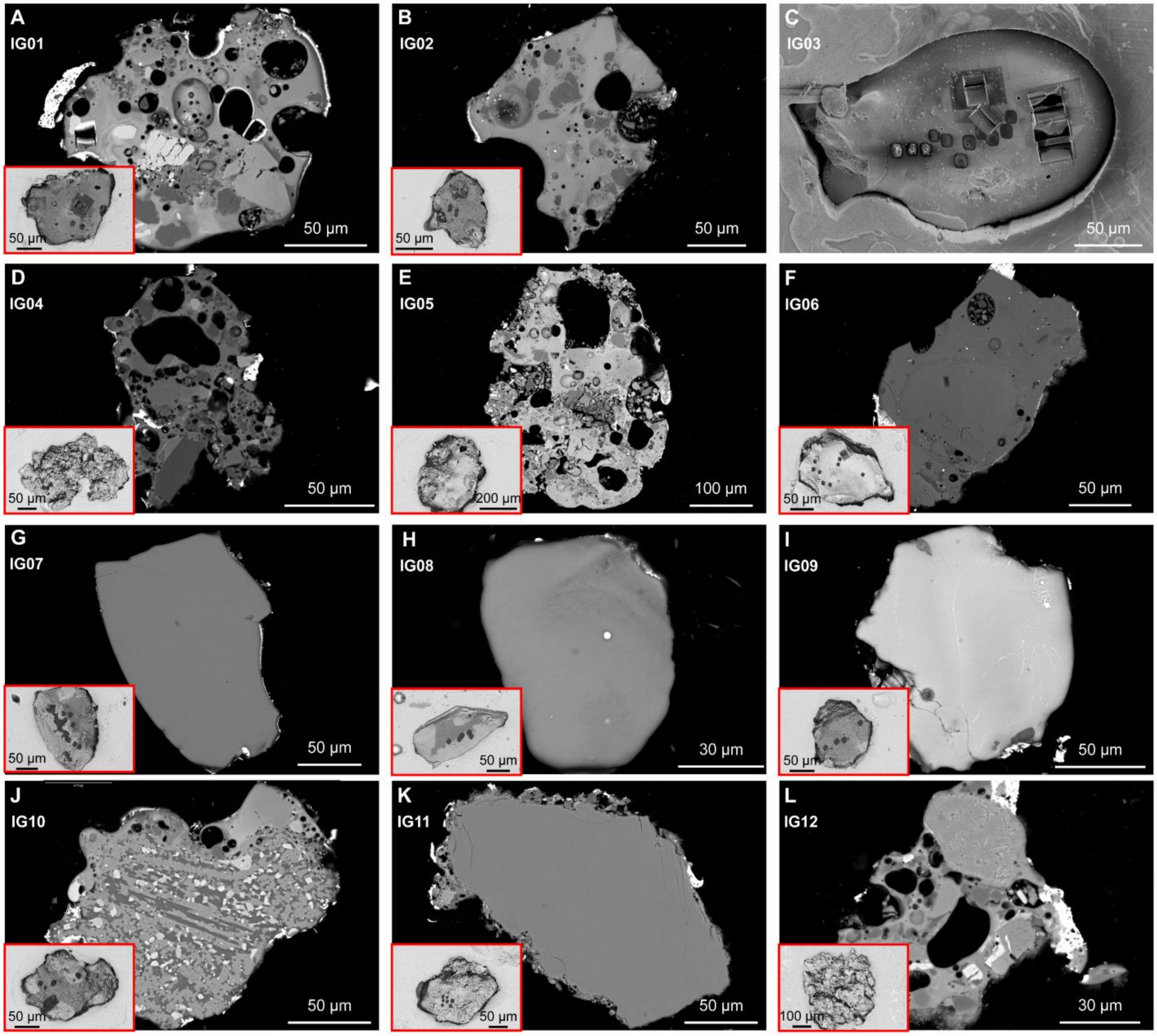

Fig. S1.

The back scattered electron images of the polished sections of Chang'e-5 impact glasses. The image to the lower left of each panel correspond to the unpolished grain. The impact glass grains in (**A-F**) are agglutinates, in (**G-I**) are pure glasses, and in (**J-L**) are amorphous coatings.

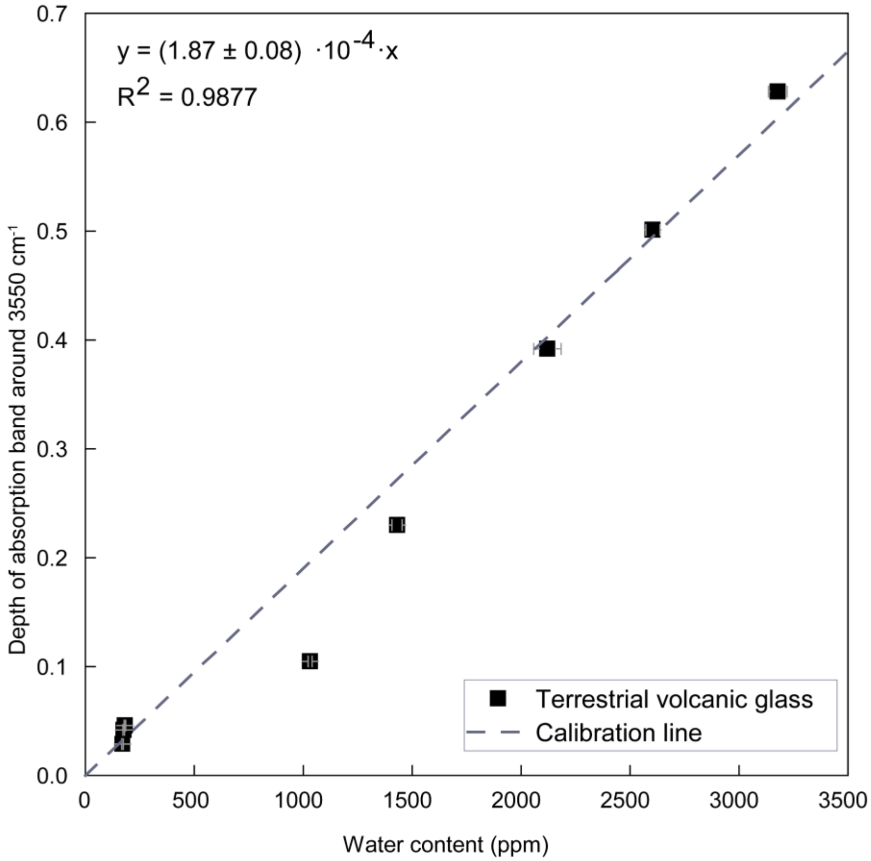

Fig. S2.

The OH/H_2O content calibration for reflectance infrared spectra based on the measurements of terrestrial volcanic glasses. The slope of the calibration line is (1.87 ± 0.08) × 10^{-4}, and the determination coefficient is 0.9877. The error bars represent 2σ.

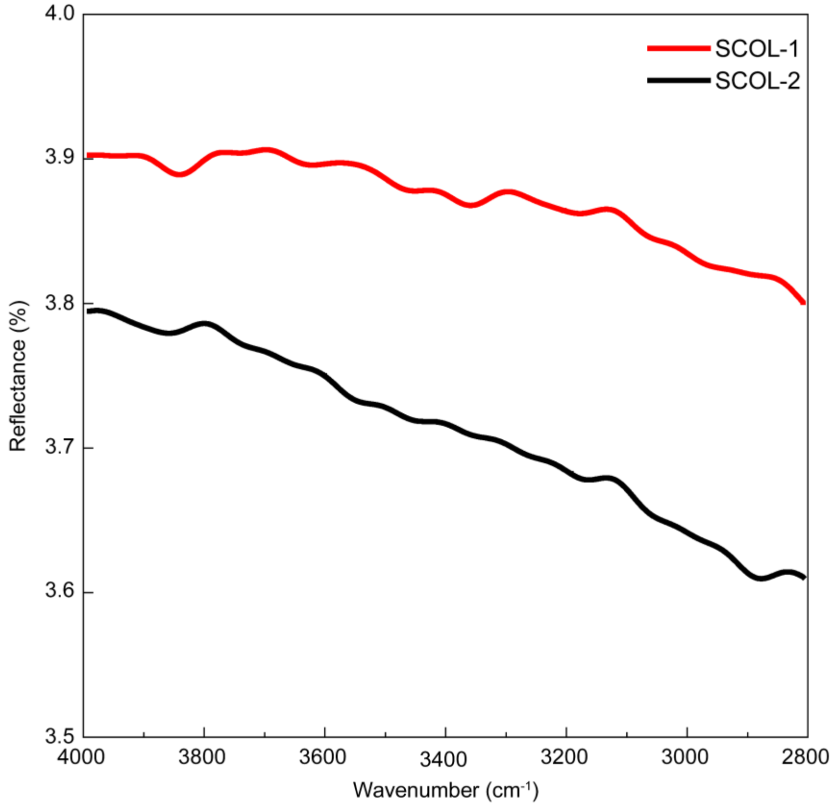

Fig. S3.

The reflectance infrared spectra of anhydrous San Carlos olivine in the range of 2800–4000 cm^{-1}.

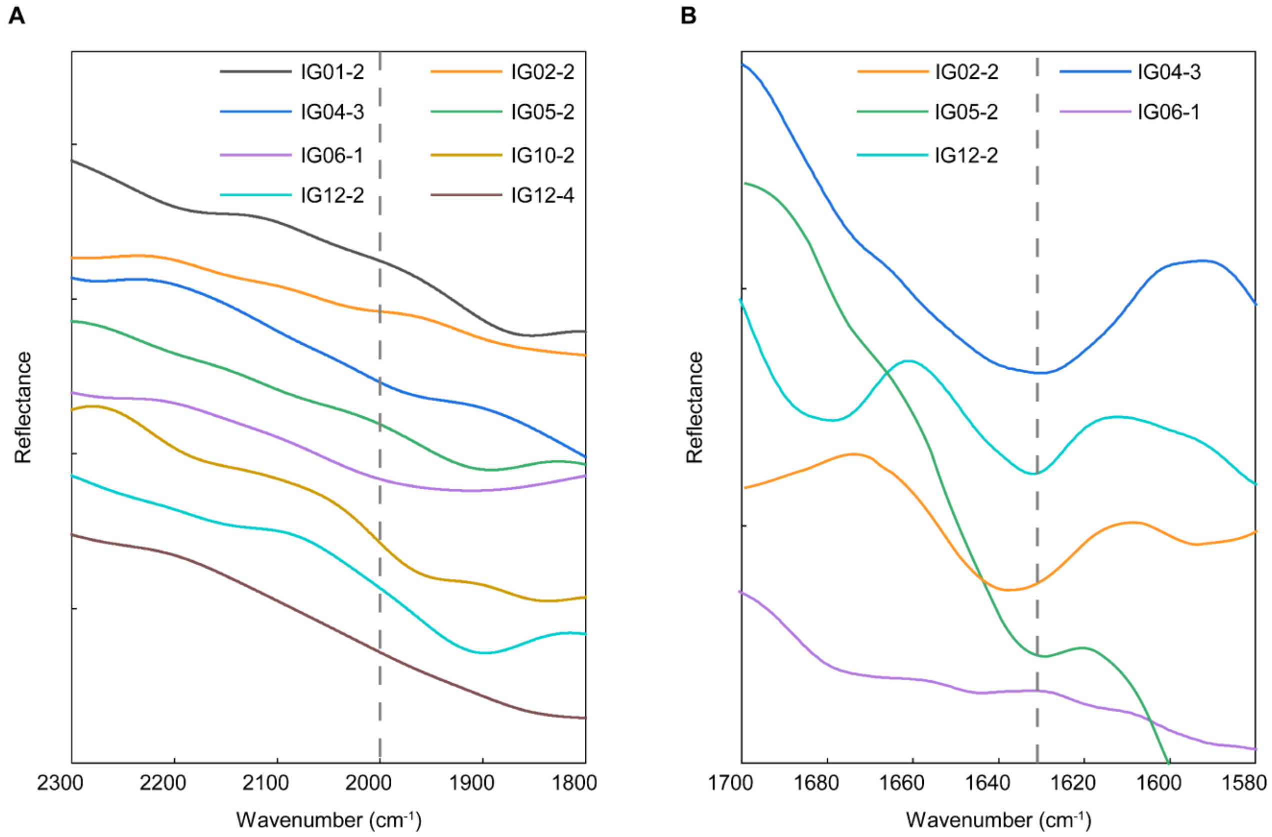

Fig. S4.

Reflectance infrared spectra of Chang'e-5 impact glasses in the range of 2300–1800 cm^{-1} (**A**) and 1700–1580 cm^{-1} (**B**). The solid lines are smoothed spectra obtained using the Fast Fourier Transform algorithm. Dashed line in (**A**) show no absorption in 2000 cm^{-1}, and dashed line in (**B**) show the absorption around 1630 cm^{-1}.

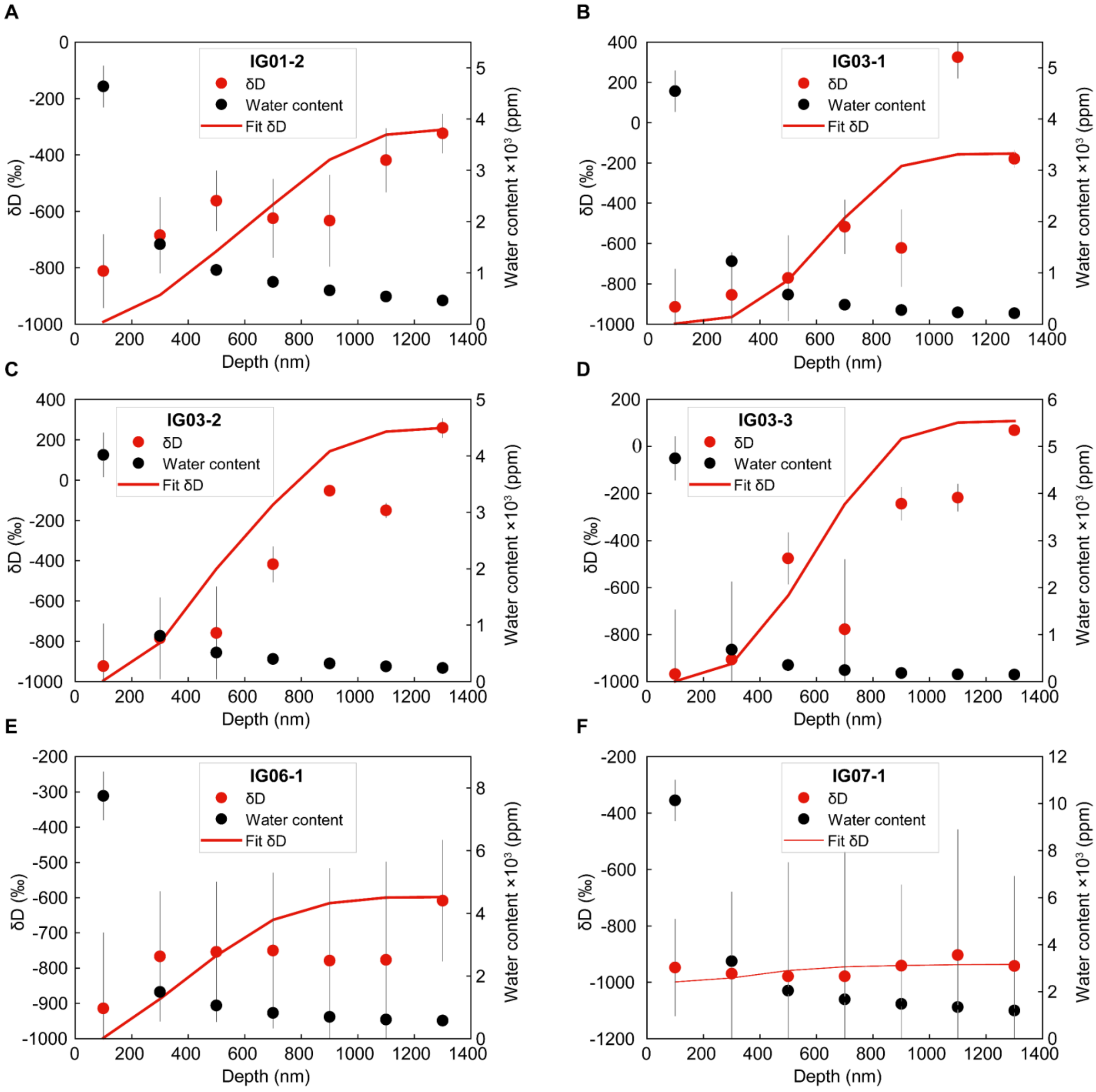

Fig. S5.

Fitting results for the distribution of water in Chang'e-5 impact glasses. The red and black circles represent the δD values and water content at different depths as measured by nanoscale secondary ion mass spectrometry. The red lines are the fitting curves of δD distribution based on a binary mixing model. All error bars (gray line) correspond to 2σ.

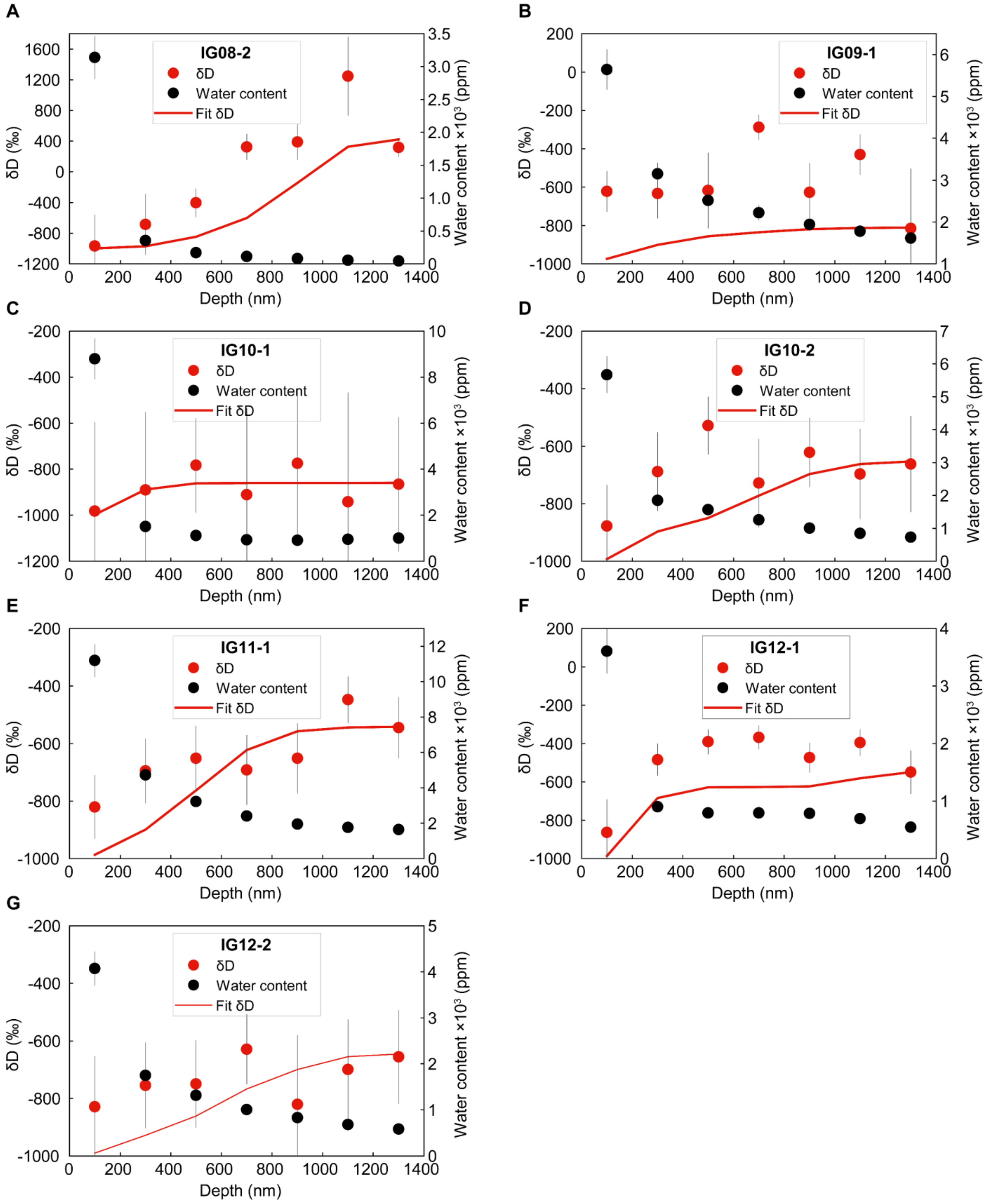

Fig. S6.

Fitting results for the distribution of water in Chang'e-5 impact glasses. The red and black circles represent the δD values and water content at different depths as measured by nanoscale secondary ion mass spectrometry. The red lines are the fitting curves of δD distribution based on a binary mixing model. All error bars (gray line) correspond to 2σ.

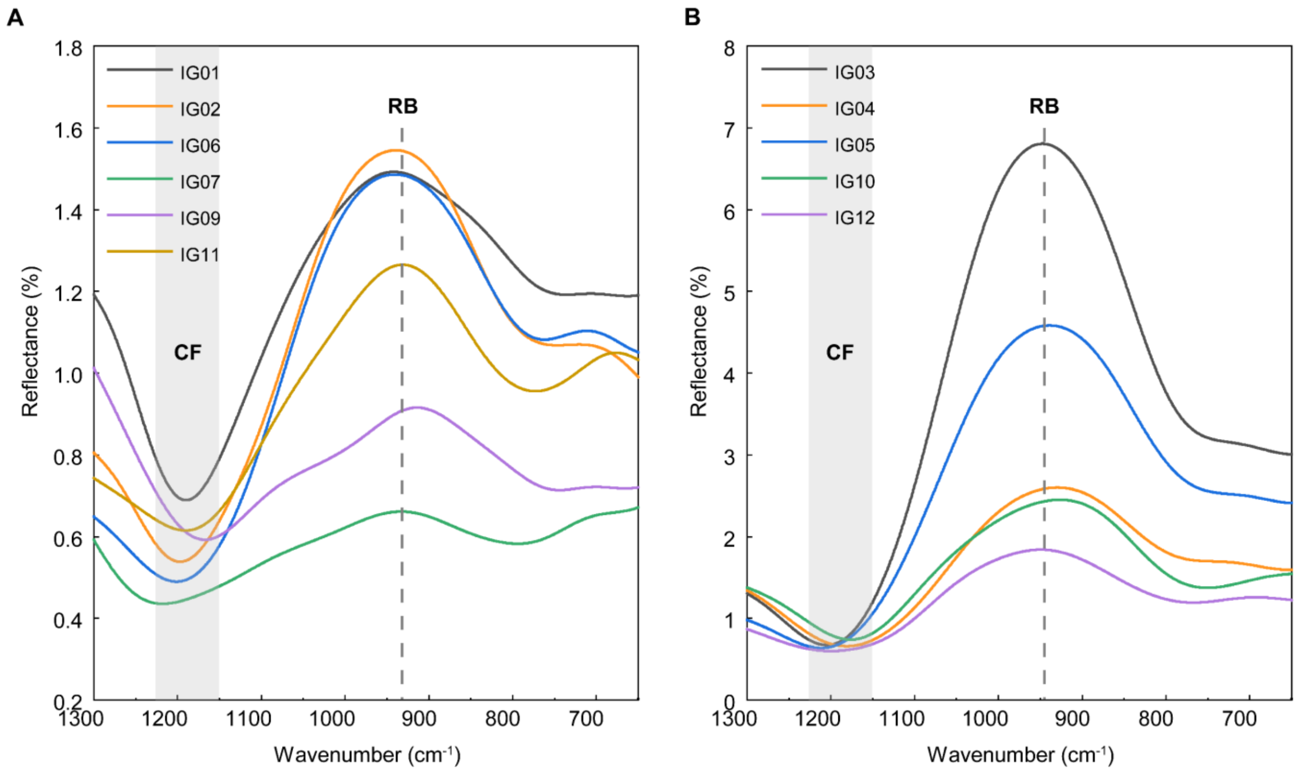

Fig. S7.

Reflectance infrared spectra in the 650–1300 cm^{-1} range for Chang'e-5 lunar impact glasses.

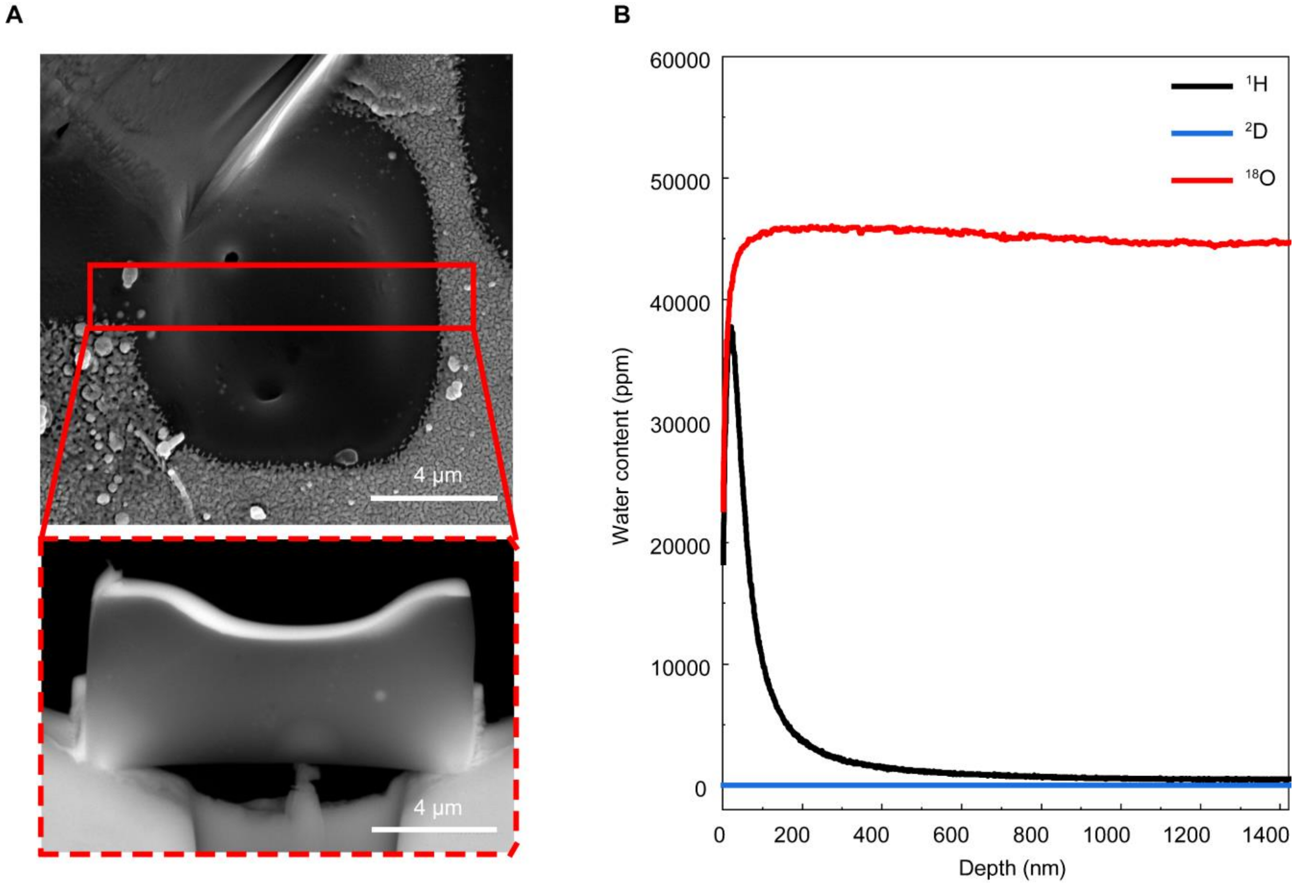

Fig. S8.

The depth of nanoscale secondary ion mass spectrometry (NanoSIMS) measurement spot. (**A**) Scanning electron microscopy (SEM) image of NanoSIMS measurement area, the green rectangle shows the focused ion beam (FIB) sampling site. (**B**) The cross section of the spot prepared by the FIB. (**C**) Examples of $^{1}H^{-}$, $^{2}D^{-}$, and $^{18}O^{-}$ counts profiles.

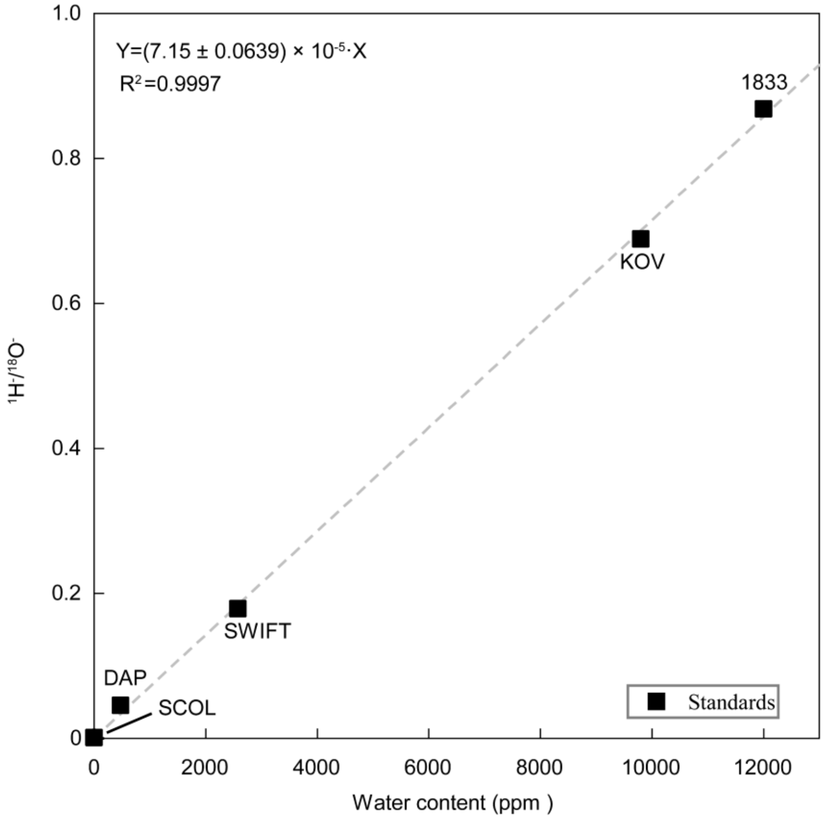

Fig. S9.

Water content calibration line for nanoscale secondary ion mass spectrometry analysis derived from the standards. The slope of the calibration line is (7.15 ± 0.06) × 10^{-5}, and the determination coefficient is 0.9997. SCOL: San Carlos olivine; DAP: Durango apatite; SWIFT: SWIFT MORB glass; KOV: Kovdor apatite; 1833: basaltic glass 1833. The analytical uncertainty is 0.50 %.

Table S1.

The bulk OH and H_2O content of Chang'e-5 impact glasses determined by reflectance infrared spectra.

Sample	Type	Bulk water content (ppm)	Bulk molecular H_2O content (ppm)
IG01-1	Agglutinate	551 ± 136	n.d.[1]
IG01-2		262 ± 65	161 ± 32
IG02-1	Agglutinate	390 ± 96	n.d.
IG02-2		433 ± 107	206 ± 41
IG03-1	Agglutinate	310 ± 76	n.d.
IG03-2		487 ± 120	n.d.
IG04-1	Agglutinate	144 ± 36	n.d.
IG04-2		551 ± 136	n.d.
IG04-3		422 ± 104	155 ± 31
IG05-1	Agglutinate	465 ± 115	n.d.
IG05-2		364 ± 90	106 ± 21
IG05-3		487 ± 120	n.d.
IG05-4		652 ± 161	n.d.
IG05-5		390 ± 96	n.d.
IG05-6		326 ± 80	n.d.
IG05-7		620 ± 153	n.d.
IG06-1	Agglutinate	283 ± 70	174 ± 35
IG07-1	Pure glass	754 ± 186	n.d.
IG07-2		781 ± 193	n.d.
IG08-1	Pure glass	n.a.[2]	n.a.
IG09-1	Pure glass	679± 167	n.d.
IG09-2		695 ± 171	n.d.
IG10-1	Amorphous coating	171 ±42	n.d.
IG10-2		492 ± 121	216 ± 43
IG11-1	Amorphous coating	326 ± 80	n.d.
IG11-2		406 ± 100	n.d.
IG12-1	Amorphous coating	235 ± 58	n.d.
IG12-2		182 ± 45	107 ± 21
IG12-3		267 ± 66	n.d.
IG12-4		487 ± 120	87 ± 17

[1]n.d. denotes that the H_2O content is too low to be detected, or the H_2O absorption is overlapped by other absorptions.

[2]n.a. denotes that the reflectance infrared spectra are not available due to the low infrared signals.

Table S2.

The water content and hydrogen isotope composition in different depths of Chang'e-5 impact glasses measured by nanoscale secondary ion mass spectrometry.

Sample	Average water content within the 1400 nm depth (ppm)	Average δD within the 1400 nm depth (‰)	Water content within the 100 nm depth (ppm)	δD within the 100 nm depth (‰)	Water content in the 1300–1400 nm depth (ppm)	δD within the 1300–1400 nm depth (‰)
IG01-1	1461 ± 124	-773 ± 104	6105 ± 502	-939 ± 234	507 ± 41	-586 ± 206
IG01-2	1389 ± 119	-691 ± 82	6500 ± 540	-862 ± 170	448 ± 36	-464 ± 154
IG03-1	1087 ± 99	-805 ± 131	6406 ± 553	-909 ± 199	210 ± 17	105 ± 30
IG03-2	928 ± 87	-740 ± 101	6376 ± 593	-934 ± 239	236 ± 19	590 ± 129
IG03-3	914 ± 92	-859 ± 122	7896 ± 716	-984 ± 399	143 ± 12	-109 ± 38
IG03-4	927 ± 84	-808 ± 108	5655 ± 490	-933 ± 244	249 ± 20	-440 ± 166
IG05-1	1281 ± 130	-843 ± 113	12550 ± 1055	-976 ± 287	174 ± 14	-271 ± 115
IG06-1	1844 ± 174	-846 ± 117	12909 ± 1190	-932 ± 306	567 ± 46	-701 ± 313
IG07-1	3017 ± 258	-953 ± 178	14087 ± 1153	-951 ± 191	1158 ± 93	-948 ± 474
IG07-2	2818 ± 241	-979 ± 172	12957 ± 1067	-988 ± 364	1201 ± 97	-962 ± 556
IG08-1	749 ± 82	-917 ± 207	6735 ± 655	-968 ± 433	37 ± 3	-239 ± 169
IG08-2	602 ± 64	-806 ± 180	5284 ± 513	-986 ± 510	41 ± 3	583 ± 292
IG09-1	2767 ± 227	-582 ± 72	7130 ± 611	-659 ± 124	1581 ± 127	-840 ± 486
IG10-1	2216 ± 207	-933 ± 163	14534 ± 1352	-984 ± 382	1017 ± 82	-762 ± 268
IG10-2	1908 ± 166	-754 ± 84	8861 ± 844	-926 ± 211	695 ± 56	-649 ± 224
IG11-1	3874 ± 324	-715 ± 75	13802 ± 1119	-840 ± 131	1628 ± 131	-648 ± 195
IG12-1	1153 ± 103	-627 ± 64	5953 ± 619	-931 ± 279	503 ± 41	-590 ± 183
IG12-2	1376 ± 116	-762 ± 89	5745 ± 510	-841 ± 189	553 ± 45	-706 ± 266

Table S3.

The continuum removed reflectance and grain size of Chang'e-5 impact glass used in the radiative transfer model. The grain size was determined using an optical microscope mounted on the Fourier transform infrared spectrometer.

Sample	Continuum removed reflectance around 1630 cm^{-1}	Grain size (μm)	H_2O content (ppm)
IG01-2	0.9682	135	161 ±32
IG02-2	0.9513	164	206 ± 41
IG04-3	0.9498	225	155 ± 31
IG05-2	0.9493	333	106 ± 21
IG06-1	0.9618	151	174 ± 35
IG10-2	0.9481	167	216 ± 43
IG12-2	0.9443	365	107 ± 21
IG12-4	0.9544	365	87 ± 17

Table S4.

The composition of Chang'e-5 impact glasses measured by electron probe microanalyzer (EPMA).

Sample	SiO_2	TiO_2	Al_2O_3	Cr_2O_3	FeO	NiO	MnO	MgO	CaO	Na_2O	K_2O	P_2O_5	SO_3	Total
IG01	40.56	5.61	8.34	0.24	22.81	0.07	0.28	12.34	8.74	0.23	0.05	0.11	0.14	99.52
IG02	41.28	6.18	8.78	0.16	23.80	0.04	0.27	7.87	9.71	0.42	0.11	0.24	0.31	99.17
IG03	45.80	4.40	13.00	n.d.[1]	17.30	1.20	n.d.	8.20	10.10	n.d.	n.d.	n.d.	n.d.	100.00
IG04	39.48	3.96	9.36	0.52	29.22	0.82	0.46	5.22	10.76	0.20	n.d.	n.d.	n.d.	100.00
IG05	35.70	8.55	10.95	0.40	23.85	0.35	0.30	7.25	10.95	1.00	0.15	0.35	n.d.	100.00
IG06	45.13	1.76	11.39	0.29	17.68	0.06	0.24	9.37	10.98	0.47	0.18	0.63	0.47	98.66
IG07	48.02	1.09	15.15	0.24	13.78	n.d.	0.17	9.44	11.32	0.09	0.06	0.02	n.d.	99.38
IG08	40.64	6.38	10.22	0.21	22.49	0.00	0.26	7.55	10.35	0.63	0.22	0.30	0.27	99.53
IG09	40.15	5.42	7.73	0.16	25.15	0.01	0.33	7.43	9.82	0.35	0.14	0.20	0.23	97.12
IG10	36.20	9.70	14.40	0.20	19.90	0.30	0.30	6.70	12.20	n.d.	n.d.	n.d.	n.d.	99.90
IG11	40.30	4.90	12.20	n.d.	22.40	0.10	0.20	6.90	11.40	0.80	0.60	n.d.	n.d.	99.80
IG12	43.09	6.38	12.53	0.21	19.22	0.06	0.22	7.04	11.76	0.67	0.17	0.31	0.56	102.22

The composition data of IG03, IG04, and IG05 were obtained by EDS mounted on SEM.
n.d.[1] denotes the content is too low to be detected.

Table S5.

Water content and depth of absorption band around 3550 cm^{-1} for terrestrial volcanic glasses. The water content was determined by nanoscale secondary ion mass spectrometry (NanoSIMS), the depth of absorption band around 3550 cm^{-1} was determined by Fourier transform infrared spectrometer (FTIR).

Sample	Water content (ppm)	Depth of absorption band around 3550 cm^{-1}
TVG-1	170 ± 4	0.029
TVG-2	176 ± 4	0.042
TVG-3	182 ± 4	0.046
TVG-4	1031 ± 9	0.105
TVG-5	1431 ± 23	0.230
TVG-6	2120 ± 62	0.392
TVG-7	2605 ± 32	0.501
TVG-8	3178 ± 42	0.628

Data S1.
Reflectance infrared spectra in the range of 2800–4000 cm^{-1} for all Chang'e-5 impact glasses.

Data S1 is provided as a separate file.
